# γδ T cells as a potential therapeutic agent for glioblastoma

**DOI:** 10.3389/fimmu.2023.1273986

**Published:** 2023-10-20

**Authors:** In Kang, Yumin Kim, Heung Kyu Lee

**Affiliations:** ^1^ Graduate School of Medical Science and Engineering, Korea Advanced Institute of Science and Technology (KAIST), Daejeon, Republic of Korea; ^2^ Department of Biological Sciences, KAIST, Daejeon, Republic of Korea

**Keywords:** glioblastoma, tumor microenvironment, γδ T cells, immunotherapy, engineering

## Abstract

Although γδ T cells comprise a small population of T cells, they perform important roles in protecting against infection and suppressing tumors. With their distinct tissue-localizing properties, combined with their various target recognition mechanisms, γδ T cells have the potential to become an effective solution for tumors that do not respond to current therapeutic procedures. One such tumor, glioblastoma (GBM), is a malignant brain tumor with the highest World Health Organization grade and therefore the worst prognosis. The immune-suppressive tumor microenvironment (TME) and immune-evasive glioma stem cells are major factors in GBM immunotherapy failure. Currently, encouraged by the strong anti-tumoral function of γδ T cells revealed at the preclinical and clinical levels, several research groups have shown progression of γδ T cell–based GBM treatment. However, several limitations still exist that block effective GBM treatment using γδ T cells. Therefore, understanding the distinct roles of γδ T cells in anti-tumor immune responses and the suppression mechanism of the GBM TME are critical for successful γδ T cell–mediated GBM therapy. In this review, we summarize the effector functions of γδ T cells in tumor immunity and discuss current advances and limitations of γδ T cell–based GBM immunotherapy. Additionally, we suggest future directions to overcome the limitations of γδ T cell–based GBM immunotherapy to achieve successful treatment of GBM.

## 1 Introduction

γδ T cells, named after their distinctive γδ T cell receptor (TCR) usage, comprise approximately 5% of all T lymphocytes (1). Similar to conventional αβ T cells, γδ T cells recognize targets and exert direct cytotoxic effector functions by secreting granzymes or perforin (2, 3) and inducing immune responses of other cells by secreting cytokines (4), thereby participating in host protection against various pathogens or tumors. Unlike αβ T cells, which recognize peptides on the major histocompatibility complex (MHC) (5), γδ T cells recognize other surface molecules (6). In humans, Vδ2^+^ T cells recognize the butyrophilin family 2A1 and 3A1 complex (BTN2A1–BTN3A1 complex) linked by phosphoantigens (7), and Vδ1^+^ T cells recognize MHC class I chain-related molecule A (8). Because these surface molecules are upregulated in the presence of infection or cellular damage (9, 10), γδTCR-mediated target recognition of γδ T cells resembles that of pattern recognition receptors. Therefore, γδ T cells function as linkers between innate and adaptive immune responses (11) and act as the first-line defense system of the body during early infection.

In addition to infection, γδ T cells have demonstrated their importance in immune responses related to tumors (12, 13). γδ T cells not only localize in peripheral organs (14) but also circulate through blood and lymphatics (15). Therefore, they play critical roles in tumor immune responses in solid cancers, such as lung (16) or colorectal cancer (17), as well as in hematopoietic malignancies (18). Particularly for solid cancers, high infiltration of γδ T cells represents a good prognosis marker (19). Therefore, many research groups have investigated γδ T cell–based immunotherapeutic procedures for cancer treatment (20, 21). Based on their diverse target recognition mechanism, a strong tendency toward activation via various types of stimulation, subsequent cytotoxic effector functions (22, 23), and MHC-independent target recognition mechanism (6), the possibility exists that γδ T cells can be effective immunotherapeutic agents that can target tumors that do not respond to current therapeutic procedures (24–26). Therefore, several research groups are investigating γδ T cell–based cancer therapy targeting various tumor models.

Glioblastoma (GBM) is a malignant tumor that occurs in the brain and is the most common yet lethal malignancy among central nervous system (CNS) tumors (27). A lack of distinctive risk factors (28) combined with nonspecific symptoms (29) make GBM difficult to diagnose in the early phase, thereby decreasing the survival rate. Many research groups have performed extensive investigations to identify an effective treatment for GBM. As a result, various mechanical (30, 31), chemical (32), and immunological (33) treatment approaches have been developed for GBM. Although some treatments have shown meaningful increases in patient survival rates (34, 35), many of those procedures did not show substantial results (26, 36, 37). Therefore, identification of novel therapeutic procedures is critical for effective treatment of GBM.

In this review, we will summarize the immunologic signatures of γδ T cells, focusing on their roles in anti-tumoral immune responses. Then, we will discuss current immunotherapeutic approaches in GBM treatment and challenges arising from the tumor microenvironment (TME) of GBM. Additionally, we will discuss current approaches to target GBM using γδ T cells and the limitations of γδ T cell–based treatments. Finally, we will suggest possible solutions to overcome those challenges in γδ T cell–based GBM immunotherapy.

## 2 γδ T cells

γδ T cells are a small subset of T cells that express the γδTCR instead of the conventional αβTCR. Even though they comprise a small population of circulating lymphocytes (38), γδ T cells localize in peripheral organs and barrier sites such as the skin, mucosal tract of the intestine or reproductive organs, and pulmonary tract (39) and comprise 15–30% of intraepithelial lymphocytes in the human gut (40). γδ T cells are further subdivided into various subsets according to their Vγ (mouse) or Vδ (human) usage, and Vγ or Vδ utilization determines their localization. In mice, γδ T cells expressing Vγ1 or Vγ4 (Tonegawa nomenclature) circulate through the bloodstream, Vγ5 is localized in the skin, Vγ6 is localized in the dermis and meninges, and Vγ7 is localized in the gut (39). In humans, Vδ2^+^ γδ T cells circulate in the blood, whereas Vδ1^+^ and Vδ3^+^ γδ T cells have resident features (13). Even though they make up a small portion of the T cell population (1), their various effector functions and distinct tissue localization make γδ T cells a first-line immune system defense mechanism by directly suppressing pathogenic infection and working as both innate and adaptive immune cells.

γδ T cells recognize various types of surface molecules, unlike conventional αβ T cells that recognize peptides loaded on the MHC. For example, human Vδ1^+^ γδ T cells recognize the CD1d molecule (41), Vγ8Vδ3^+^ T cells recognize stress-induced annexin A2 (42), and Vγ9Vδ1^+^ T cells recognize ephrin type-A receptor 2 induction by AMP-activated protein kinase (43). In addition to these tissue-localizing human γδ T cells, Vγ9Vδ2^+^ T cells circulating in the peripheral blood recognize the BTN2A1-BTN3A1 complex in the presence of phosphoantigens (7, 44). Because γδTCRs recognize stress-induced molecules expressed on the target cell surface, recognition of γδTCRs resembles that of pattern recognition receptors (45). Therefore, γδ T cells possess invariant or semi-variant signatures, unlike αβ T cells, which have to recognize various peptides; therefore, TCR diversity is critical (46). In addition to the γδTCR, γδ T cells recognize a broad spectrum of surface molecules via NK receptors (NKRs) and exert effector functions synergistically with γδTCR ligation (47). In addition to γδTCR and NKR-mediated target recognition and effector function, γδ T cells may exert a cytolytic function via death ligands (Fas-ligand or TRAIL) (48, 49). With these multi-faceted target recognition mechanisms, γδ T cells play important roles in the first-line protection of various tissues (50, 51).

γδ T cells exert multiple effector functions and share those effector functions with conventional αβ T cells. For example, γδ T cells lyse target cells by granzyme and perforin production (52), similar to cytotoxic CD8^+^ T cells. Additionally, γδ T cells secrete various cytokines, including IFNγ and TNFα, demonstrating that γδ T cells can modulate the immune system through cytokine production (53). Furthermore, similar to effector CD4^+^ T cells, γδ T cells polarize into distinct subtypes and concomitantly produce cytokines that affect the surrounding immune microenvironment. Among murine γδ T cells, IL-17-producing γδ T cells and IFN-γ-producing γδ T cells differentially develop in the thymus (54) and perform distinct roles (55, 56). In contrast, human Vγ9Vδ2^+^ T cells show functional plasticity (57, 58) according to their exposure to cytokines during TCR stimulation. This functional plasticity of γδ T cells makes them multi-faceted effectors that exert both protective and damaging effects in disease conditions, including cancers (1).

### 2.1 Roles of γδ T cells in tumor suppression

Among the multi-faceted roles of γδ T cells in tumor conditions, tumor-suppressive roles of γδ T cells have been extensively studied by many research groups because of their high cytotoxicity, multipotent effector function, and unique tissue localization, along with the fact that their presence is a positive prognostic marker for all types of solid tumors (19). In a mouse model of prostate cancer, Liu et al. showed that knockout of γδ T cells resulted in extensive tumor growth, and adoptive transfer of γδ T cells significantly reduced tumor burden (59). Moreover, γδ T cells showed superior tumor control compared with the same number of conventional αβ T cells, demonstrating that γδ T cells have better tumor suppression and target-lysing abilities than conventional T cells without tumor specificity. Similarly, in the colorectal cancer model induced by azoxymethane, mice lacking γδ T cells had a higher tumor incidence than those lacking αβ T cells, demonstrating that γδ T cells can act as a primary tumor suppressor (60). Also, in chemically induced skin cancer, knockout of γδ T cells significantly increased tumor growth, whereas depletion of αβ T cells did not affect tumor formation and growth. Therefore, γδ T cells act as tumor suppressors in various organs, including the skin and colon.

**Figure 1:**
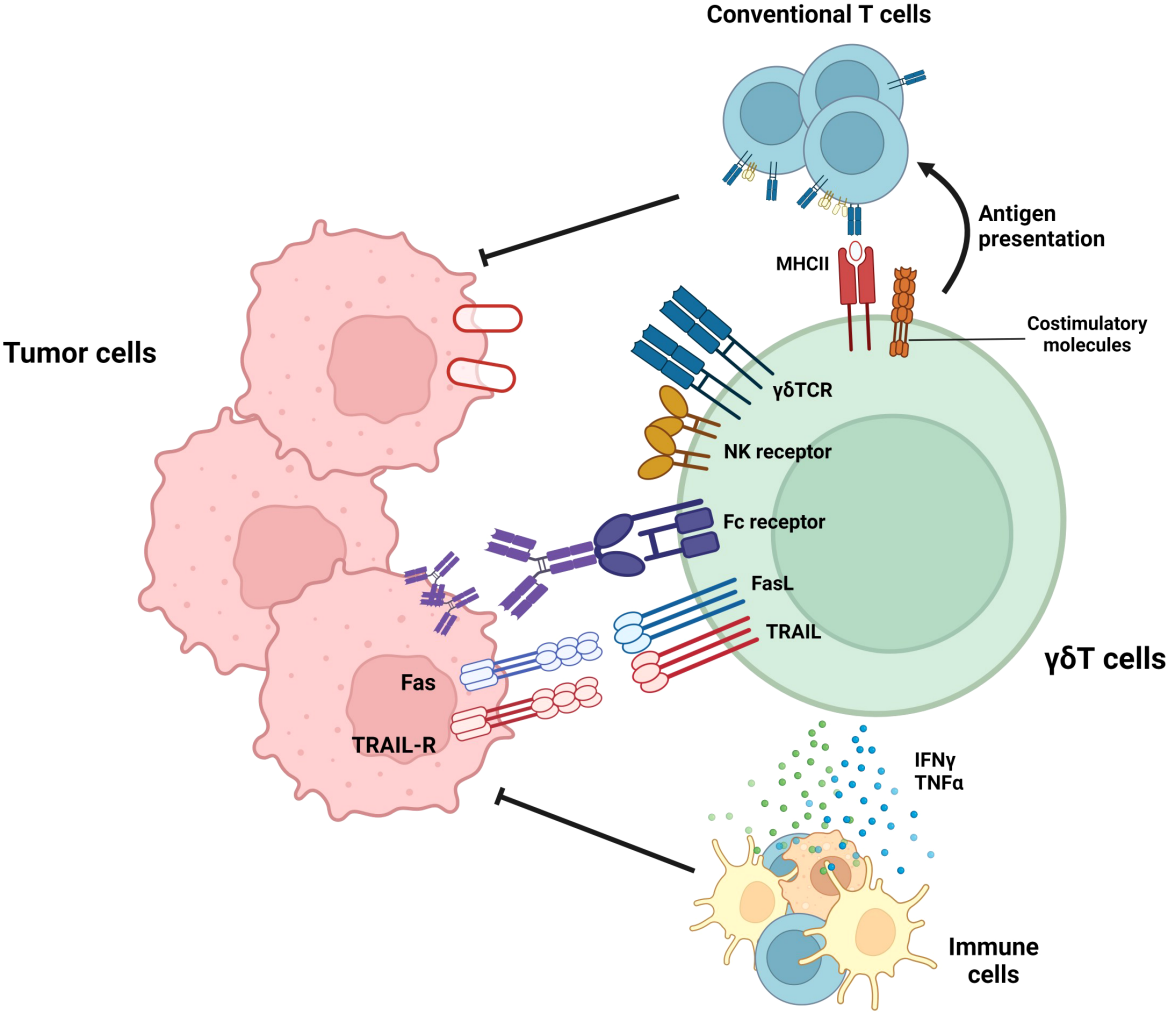
Roles of γδ T cells in tumor suppression. γδ T cells exert anti-tumoral immune responses by diverse mechanisms. By recognizing target molecules via γδTCR and NKG2D, γδ T cells can lyse tumor cells. In addition, Vδ2+ γδ T cells can eliminate tumor cells by antibody-dependent cell-mediated cytotoxicity (ADCC) in a CD16-dependent manner. Furthermore, γδ T cells can suppress tumor cells by death ligands, such as TRAIL or Fas ligands. In addition to these direct killings, γδ T cells can indirectly suppress tumor cells by activating T cells via working as antigen-presenting cells (APCs) or facilitating other immune cells via pro-inflammatory cytokine secretion.

In addition to the anti-tumor functional studies of mouse γδ T cells, human γδ T cells have demonstrated anti-tumor function. **Figure 1** summarizes the anti-tumoral effector functions of γδ T cells. In case of human Vδ2^+^ γδ T cells, which bind to BTN2A1-BTN3A1 complex in the presence of phosphoantigens, can exert anti-tumoral functions (61). In addition to γδTCR-mediated cytotoxicity, Vδ2^+^ γδ T cells also exert cytolytic function via NKG2D-mediated target recognition (62). Furthermore, human Vδ2^+^ γδ T cells-but not Vδ1^+^ γδ T cells-can eliminate tumor cells by antibody-dependent cell-mediated cytotoxicity, and the cytotoxicity was proportionate to CD16 upregulation (63).

γδ T cells regulate not only tumor growth via cytotoxic effector function but also other immune cells. Unlike αβ T cells, activated γδ T cells upregulate MHC-II and other co-stimulatory molecules (CD40, CD80, and CD86) and can activate conventional T cells (64). In addition to their high cytotoxicity, γδ T cells can kill tumor cells and present the tumor antigen to conventional T cells, thereby facilitating systemic immune response against tumor cells. Moreover, γδ T cells can augment the functionality of dendritic cells, thereby facilitating antigen presentation and priming of conventional T cells (65). In summary, γδ T cells can efficiently lyse tumor cells, spread the tumor antigen, and facilitate adaptive and systemic immune responses against tumors. Therefore, γδ T cells can be a promising solution to improve current anti-tumor immunotherapy. Thus, many research groups have expanded the utilization of γδ T cells by investigating their roles and effector functions in various types of cancers and have attempted to treat cancers that do not respond to current therapeutic procedures such as immune checkpoint inhibitors (24, 25, 66, 67). One example of these cancer types is GBM, a malignant brain cancer that shows limited therapeutic responses to immune checkpoint inhibitors (26). Recently, several research groups demonstrated the importance of γδ T cells in glioma suppression (68, 69). Therefore, γδ T cells have the potential to become an effective therapeutic agent for GBM. However, several limitations exist that suppress the optimal effector function of γδ T cells in the GBM TME (68, 70–72). Therefore, the general background and current therapeutic procedures targeting GBM will subsequently be discussed. Furthermore, current advances and limitations in γδ T cell–mediated GBM treatment will be investigated. Finally, we will suggest several methodologies to overcome the limitations of γδ T cells in GBM immunotherapy.

## 3 GBM: Epidemiology and classification

GBM is a malignant brain tumor that is classified as WHO grade IV. Annually, approximately 10 out of every 100,000 people are diagnosed with GBM (73). Though the overall incidence is relatively low compared with other types of cancers, GBM is the most common malignant tumor occurring in the CNS (74) and has one of the worst prognoses of all cancer types. GBM patients survive less than 1 year without treatment, and the 5-year survival rate is less than 10% even with intensive care (34). GBM typically occurs in old adults, but it can also occur in children (75). GBM more commonly occurs in male patients than in female patients (76), and female GBM patients have better responses to standard treatment (radiotherapy + temozolomide) (77). Several studies of the risk factors of GBM have revealed that high-dose ionizing radiation (78–80) and rare genetic disorders, such as neurofibromatosis (81), increase GBM incidence. However, other risk factors, including smoking, alcohol uptake, and exposure to pesticides or steroidal hormones were not correlated with GBM onset (28). Common symptoms of GBM are headache, seizures, and cognitive and behavioral impairment (29). Because these symptoms are nonspecific, patients usually miss the opportunity for early therapeutic intervention.

Recent research revealed that GBM starts in the subventricular zone of the brain and spreads to the cortex (82). GBM originates from three cell types: neural stem cells (NSCs), NSC-derived astrocytes, and oligodendrocyte precursor cells. Among these, NSC and NSC-derived astrocytes are the more frequent cells of origin that induce GBM (83). Moreover, GBM consists of glioma stem cells (GSCs), which develop into a heterogenous cell population responsible for increasing GBM tumor burden (84). GSCs contribute to GBM’s resistance to chemoradiotherapy and high recurrence rate (85).

Current studies on molecular and genetic signatures have enabled researchers to classify GBM into various subtypes. According to the WHO classification, IDH-wildtype GBM is characterized by *TERT* promoter mutation, epidermal growth factor receptor (*EGFR*) amplification, and a combination of chromosome 7 duplication and chromosome 10 loss (86). Using gene expression patterns, researchers further classified GBM into four different subtypes: proneural, neural, mesenchymal, and classical (87, 88). Not only do these subtypes express different morphological signatures and distinct genes (89), but they also show different susceptibility toward therapeutics. Classical subtypes, which possess a TP53 mutation, show susceptibility to radiotherapy and concurrent chemotherapy with temozolomide (90). By contrast, the mesenchymal GBM subtype shows resistance to radiotherapy and chemotherapy (91, 92). Although GBM cells are classified into various subtypes, the subtypes are not stable because transitions between subtypes frequently occur, most commonly to the mesenchymal subtype from other subtypes. Ionizing radiation (91, 93) from radiotherapy and hypoxic stress (94) that arises during tumor progression instigate this transition to the mesenchymal subtype. In addition to the four subtype-based GBM classifications, epigenetic signatures can differentiate GBM types. The methylation status of the O (6)-methylguanine-DNA methyltransferase (MGMT) promoter can be used to categorize GBM tumor cells as *MGMT* promoter methylated or unmethylated. The classification by *MGMT* promoter methylation is important for GBM patient prognosis because MGMT-expressing GBM cells are more resistant to DNA alkylating agents, such as temozolomide. Therefore, those patients with *MGMT* promoter methylation in GBM tumor cells respond better to temozolomide treatment and live longer (95).

## 4 Therapeutic procedures targeting GBM

Currently, the Stupp protocol is the standard care for GBM. The protocol reduces tumor burden by resecting GBM to the extent feasible followed by radiotherapy and concomitant chemotherapy using temozolomide, a DNA alkylating agent administered orally or intravenously (96). Although this therapeutic approach improved overall survival, GBM still has a poor prognosis due to the recurrence of tumors after treatment, which leads to a lower survival rate. This high recurrence rate is a result of the intrinsic characteristics of GBM, the unique anatomical and immunological features of the brain, and the limitations of the current treatment procedures. First, GBM cancer cells undergo a mesenchymal transition during tumor progression or due to radiation therapy. This mesenchymal transition is driven by hypoxia-inducible factors (97), and the high hypoxic signature of GBM can promote mesenchymal transition. Cancer cells exhibiting a mesenchymal signature can invade through the surrounding normal brain tissue (98), which makes it difficult to determine the boundary of the GBM and renders complete resection of the tumor impossible. Furthermore, GSCs in brain tumors undergo self-renewal and differentiation (99), thereby contributing to tumor recurrence if not completely removed (100). In addition, the brain is protected by the blood–brain barrier (BBB), which hinders active involvement of the external immune system (101). As a result, brain tumors are classified as immunologically cold cancers with limited infiltration of lymphoid cells, particularly T cells (102). These characteristics lead to the ineffectiveness of various therapeutic procedures in the context of GBM treatment (26), even though those procedures have proven effective in other types of cancers (103). Moreover, brain-residing microglia (104) and neurons (105) maintain an anti-inflammatory immune environment, which hinders a robust tumor-suppressive immune response even when immune cells infiltrate the GBM. Lastly, the standard of care for GBM patients does not use target-specific therapeutic agents and may lead to off-target toxicity in the surrounding normal cells. GBM surgical resection leads to the loss of normal tissues surrounding the tumor, and radiation therapy can deplete brain immune cells or trigger mutations in normal brain tissue, potentially leading to the initiation of new tumor foci. It can also promote the mesenchymal transition of existing cancer cells, increasing resistance to drugs and radiation therapy (106). Temozolomide can affect normal cells as well, including immune cells. Most importantly, brain tumors with an unmethylated *MGMT* promoter exhibit resistance to temozolomide (107). In 2014, it was discovered that the addition of anti–vascular endothelial growth factor therapy, which inhibits angiogenesis, had a synergistic effect with conventional treatment methods in recurrent gliomas. However, the improvement in patient survival resulting from this combination therapy was found to be modest (108). Similarly, although the utilization of a novel treatment method, called tumor-treating fields (35), has led to a meaningful improvement in overall survival in brain tumor patients, overall patient survival rates remain low (109). To overcome the current limitations of brain tumor therapy, it is crucial to devise novel therapeutic approaches that not only effectively remove tumors but also facilitate the involvement of the immune system to prevent tumor recurrence. Consequently, research has emphasized the necessity of immunotherapy, a treatment modality that focuses on enhancing the immune response against brain tumors.

### 4.1 Immunotherapeutic approaches targeting GBM

The brain has historically been considered an immunologically privileged site, where immune activation is suppressed by the presence of the BBB and the immunosuppressive microenvironment (110). However, it has been revealed that the brain, like other organs, also possesses draining lymph nodes (111). Additionally, brain tumors with a higher infiltration of T cells are associated with better patient survival (112). This discovery suggests that immune surveillance also occurs in the brain, underscoring the significance of immune cell involvement in brain tumor therapy. Because various immunotherapies have proven effective in treating various types of cancers, there have been efforts to apply these immune-based treatments to GBM as well. These endeavors can be broadly categorized into four main approaches: immune checkpoint inhibitors, oncolytic viruses, vaccination, and cell-based therapies. Despite their success in clinical trials for several types of tumors (113–115), these immunotherapies have not achieved meaningful success in GBM patients (26, 36, 116). Hence, it is crucial for future advancements in brain tumor therapy to investigate why conventional immunotherapies have not been effective in GBM treatment and propose treatment strategies to overcome these limitations.

### 4.2 Challenges in using current immunotherapies to treat GBM

**Figure 2:**
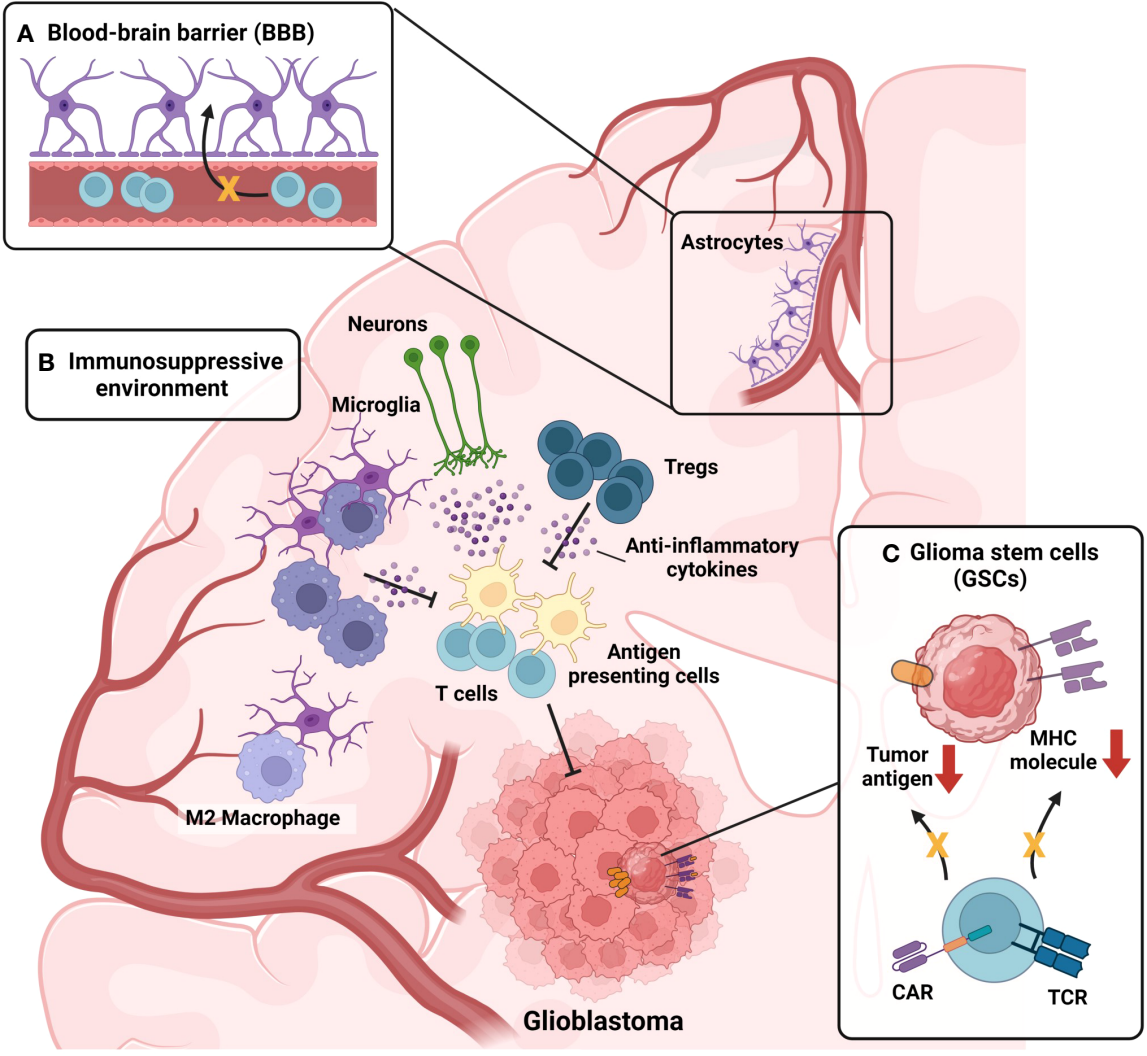
Challenges in current immunotherapy for GBM. **(A)** Presence of the Blood-brain barrier (BBB) act as a limiting factor for GBM immunotherapy. BBB hinders the infiltration of drugs and immune cells into brain parenchyma. High levels of anti-inflammatory cytokines present in brain parenchyma under GBM condition further suppress the breach of immune cells through the glial limitans of BBB. **(B)** Immunosuppressive microenvironment of GBM suppresses tumor-suppressive immune responses of infiltrated immune cells. Monocytes infiltrated into the GBM tumor microenvironment (TME) are skewed toward anti-inflammatory M2 phenotype, becoming M2-polarized bone-marrow-derived macrophages (BMDMs). M2-polarized BMDMs further strengthen anti-inflammatory TME by secreting anti-inflammatory cytokines. Not only myeloid cells but also, lymphoid cells, sustain the anti-inflammatory TME of GBM. Regulatory T cells (Tregs) are present in GBM TME, participating in the formation of immunosuppressive TME. In addition, microglia and neurons also participate in the formation of the anti-inflammatory immune environment of the brain, by secreting anti-inflammatory cytokines. **(C)** Intrinsic characteristics of glioma stem cells (GSCs) also contribute to the resistance of immunotherapies. By activation of the Wnt-β-catenin pathway, GSCs downregulate the expression of MHC-I expression, evading T cell immunosurveillance. Also, GSCs can evade chimeric-antigen receptors (CAR)-mediated immunosurveillance in the case of CAR-T treatment by downregulating the antigen targeted by CAR.

The lack of efficacy of conventional immunotherapies for GBM is attributed to both the characteristics of the brain and the unique features of GBM. **Figure 2** represents the characteristics of the brain and GBM TME that participate in the suppression of GBM immunotherapy. First, the brain is not directly connected to the bloodstream due to the presence of the BBB (**Figure 2A**). Although the BBB plays a protective role by distinguishing the brain from the periphery under normal conditions, it can hinder drug delivery and immune cell infiltration in pathological conditions, such as GBM. In cases of neuroinflammation, such as experimental autoimmune encephalomyelitis, the glial limitans of the BBB become leaky, which allows peripheral immune cells to reach the brain parenchyma (117). In the context of GBM, the influx of immune cells is inhibited due to high levels of anti-inflammatory cytokines, which suppress the migration of peripheral immune cells to the brain parenchyma (70). Indeed, reports have indicated that the BBB remains intact even in the presence of brain tumors (118), which suggests that the BBB may limit the effectiveness of immunotherapy in GBM. The production of anti-inflammatory cytokines by normal brain tissue (71) suppresses not only immune cell infiltration but also the effector function of infiltrated immune cells (**Figure 2B**). Infiltration of lymphocytes is reduced in GBM, whereas myeloid cells, especially bone marrow-derived macrophages and monocytes, are highly abundant (119). In GBM, bone marrow-derived macrophages are polarized toward an M2 phenotype in response to the anti-inflammatory brain microenvironment. These M2 macrophages play a critical role in establishing and sustaining the anti-inflammatory microenvironment of GBM, leading to the suppression of immune cell function and ultimately contributing to a decrease in patient survival rates (120). In the GBM anti-inflammatory immune environment, regulatory T cells (Tregs) are well known for their ability to suppress the functions of effector T cells and antigen-presenting cells (121). Recurrent GBM patients have a higher proportion of Tregs among their immune cells, and this elevated Treg ratio is associated with lower patient survival rates (122). Not only immune cells but also microglia (104) and neurons (105), which reside in brain parenchyma from the homeostatic condition, participate in the formation of the anti-inflammatory immune environment of the brain (**Figure 2B**). In normal conditions, that immunosuppression is protective for brain homeostasis, but in tumor conditions, that immunosuppression hinders a robust tumor-suppressive immune response against the GBM. In addition to the anti-inflammatory immune environment, the inherent characteristics of GBM cancer cells also contribute to resistance to immunotherapies. GSCs downregulate major histocompatibility complex-I (MHC-I) and antigen-processing machinery via activation of the Wnt/β-catenin pathway, thereby leading to evasion from T cell-mediated immunosurveillance (123) (**Figure 2C**). In addition, GBM shows high intra-tumoral heterogeneity (124, 125); therefore, single target–based chimeric antigen receptor (CAR)-T cell therapy or vaccination cannot eliminate tumor cells that do not express the target antigen or peptides (**Figure 2C**). Because numerous factors act as obstacles to the effectiveness of current immunotherapeutic procedures, novel therapeutic approaches are required to overcome these hurdles, and γδ T cell-mediated immunotherapy can be the solution. From now on, we will focus on the GBM immunotherapy utilizing γδ T cells, on their advances and facing limitations. Then, we will suggest several methodologies to overcome the limitations.

## 5 γδ T cells in GBM immunotherapy

### 5.1 Current advances in γδ T cell-mediated GBM immunotherapy

Encouraged by their strong anti-tumor function in preclinical and clinical research, the functionality of γδ T cells in GBM has been studied at both the preclinical and clinical levels. Park et al. demonstrated that enrichment of γδ T cells is a positive prognostic marker for survival in both mice and humans. However, γδ T cell functions in the TME are suppressed by severe hypoxia. As a result, γδ T cells downregulate NKG2D expression, which suppresses their target recognition and effector functions. Therefore, resolving tumor hypoxia through metformin treatment restored γδ T cell functionality (68). Lee et al. revealed that Vγ9Jγ2-Vδ2 T cells preferentially infiltrate the GBM TME, suggesting that human γδ T cells mediate tumor suppression *in-vivo* (69). In an *in-vitro* cytotoxicity model, human peripheral blood mononuclear cell (PBMC)-derived γδ T cells showed higher cytotoxicity on the U251MG human glioma cell line compared with αβ T cells. In addition, human PBMC-derived γδ T cells did not show cytotoxicity to non-tumor cells, such as primary human astrocytes (126). The effectiveness of γδ T cells in GBM therapy is also revealed by their ability to suppress GSCs, which are responsible for tumor initiation, maintenance, metastasis, and resistance to standard therapy (127). GSCs evade immune surveillance via MHC class I downregulation and antigen-processing machinery, thereby evading the CD8 T cell-mediated immune response (123). Despite this, γδ T cells can target GSCs. Jarry et al. injected primary GBM cells rich in GSCs (~25%) into the brains of immunocompetent (NSG) mice. Then, they injected bromohydrin pyrophosphate–activated human Vγ9Vδ2^+^ T cells into the tumor site, which successfully controlled tumor growth in combination with zoledronate (128). The superior targeting ability of γδ T cells also originated from their low activation threshold. CD8 T cells cannot be activated by NKG2D alone and require TCR signaling (129), whereas γδ T cells can be activated by NKG2D alone (23). Therefore, γδ T cells are more readily activated in the absence of TCR engagement, making it difficult for tumor cells to evade the surveillance of γδ T cells. Encouraged by those effector functions, Choi et al. showed that intra-tumoral transfer of human Vγ9Vδ2^+^ T cells significantly improved survival in mice that were injected with the U87 human glioma cell line. When analyzed by co-culturing γδ T cells with a human glioma patient-derived sample, Vγ9Vδ2^+^ T cells showed DNAM1-mediated cytotoxicity, suggesting the possible mechanism of the γδ T cell–mediated tumoricidal effector function against GBM (130).

However, clinical studies using γδ T cells have shown disappointing results in various tumor settings, and only one currently recruiting clinical trial was designed to target GBM with γδ T cells (ClinicalTrials.gov Identifier: NCT04165941). γδ T cells did not cause severe toxicity after *in-vitro* expansion and subsequent adoptive transfer (131, 132), but their therapeutic effect was moderate (21). Even though γδ T cells are promising immunotherapy to treat cancers, including GBM, several obstacles must be overcome to fully utilize γδ T cells in the clinical setting.

### 5.2 Limitations of γδ T cells in GBM immunotherapy

**Figure 3:**
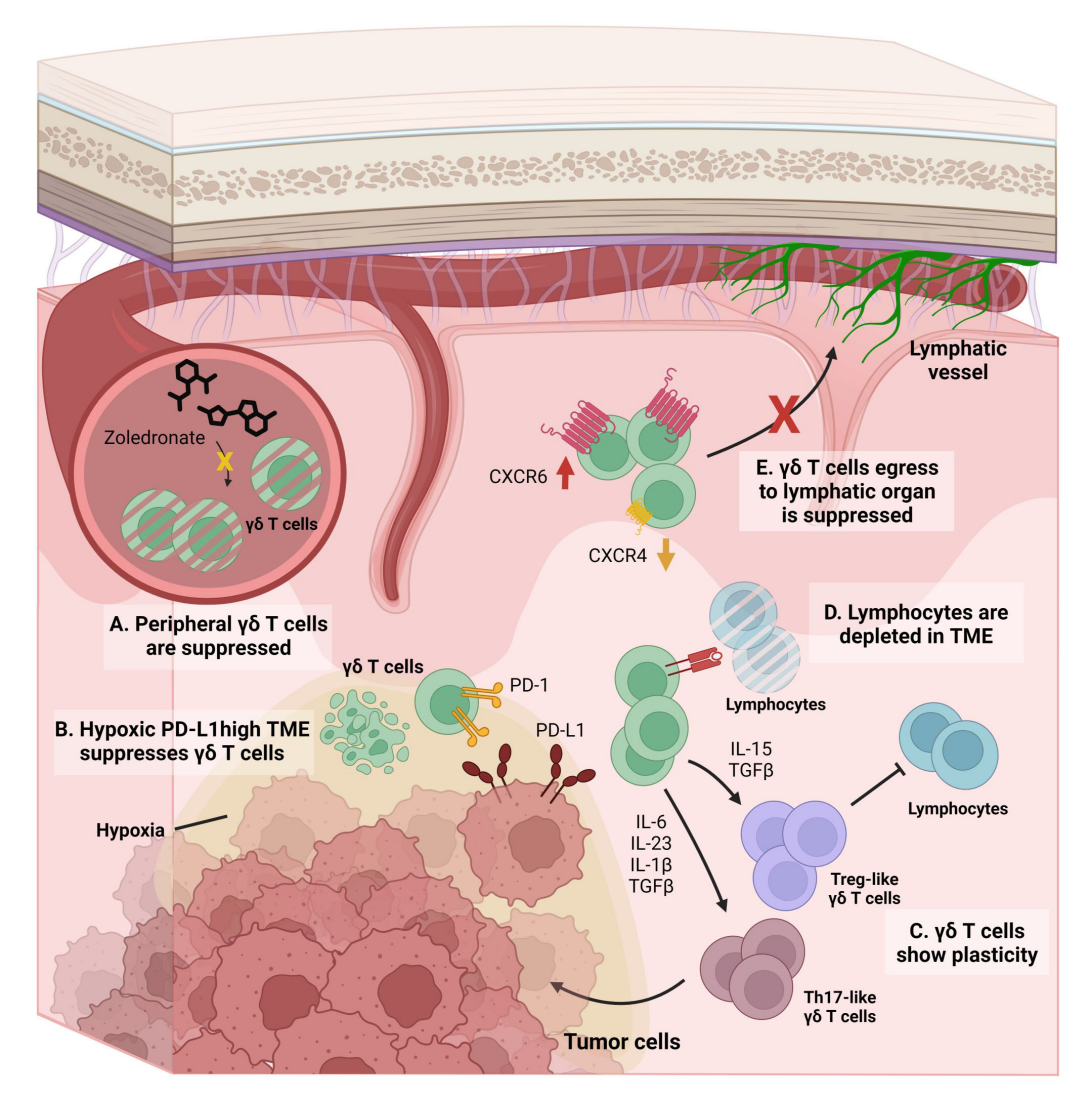
Limiting factors on γδ T cell-based GBM immunotherapy. **(A)** In the peripheral blood of GBM patients, γδ T cells are significantly decreased. Therefore, expansion of γδ T cells via *in-vivo* administration of zoledronates does not fit for GBM treatment. **(B)** Immune-suppressive microenvironment of GBM suppresses the optimal function of γδ T cells. For example, hypoxia present in GBM TME suppresses the tumoricidal function of γδ T cells by downregulating NKG2D expression on γδ T cells. In addition, PD-L1-enriched GBM TME suppresses γδ T cells by ligation with PD-1 expressed on γδ T cells. γδ T cells target GBM tumor cells in a TCR-dependent manner and express PD-1. In this condition, PD-L1-enriched GBM TME is detrimental to the optimal activation and function of γδ T cells. **(C)** Plasticity of γδ T cells can act as a detrimental factor for anti-tumoral functionality of γδ T cells. In the presence of IL-15 and TGF-β, γδ T cells skew toward the Treg-like population, thereby conspiring with other anti-inflammatory immune cells and suppressing tumoricidal functionality. Likewise, in the presence of IL-6, IL-23, IL-1β and TGF-β, γδ T cells can act as Th17-like cells, thereby facilitating tumor growth. **(D)** Lymphocyte-depleted signature of GBM TME also dampens the optimal functionality of γδ T cells. Even though γδ T cells can activate T cells by their antigen-presenting functionality, they cannot initiate T cell-mediated anti-tumoral responses due to the scarcity of lymphocytes in GBM TME. **(E)** Even though γδ T cells can phagocytose and act as antigen-presenting cells (APCs), they cannot migrate and work in draining lymph nodes, due to downregulation of CXCR4 and concomitant CXCR6 upregulation induced by TCR stimulation.

Several limitations may explain the modest effect of γδ T cells on tumor control in clinical settings, including GBM (**Figure 3**). Regarding *in-vivo* zoledronate administration, because Vδ2^+^ γδ T cells are significantly reduced in the peripheral blood of GBM patients (133), γδ T cell expansion does not produce the expected amount of cells (**Figure 3A**). Therefore, the number of expanded γδ T cells *in-vivo* is not sufficient to fully control the tumor, even after expansion by zoledronic acid treatment (133). Next, the GBM TME can suppress the effector function of γδ T cells (**Figure 3B**) (68, 72). As demonstrated by Park et al., a hypoxic TME not only induces γδ T cell exhaustion but can also make γδ T cells ineffective at targeting tumor cells (68). Therefore, γδ T cells may not target tumor cells *in-vivo* even though they could lyse tumor cells *in-vitro*. Also, the TME can have deleterious effects on γδ T cells. GBM expresses PD-L1, and PD-L1 expression is negatively correlated with patient survival (72). Because T cells upregulate PD-1 upon TCR stimulation (134), γδ T cells that have infiltrated the brain and sensed tumor cells may also express high levels of PD-1. Therefore, γδ T cells may be functionally impaired and cannot exert cytotoxic effector functions even though they expanded and infiltrated the GBM TME (**Figure 3B**). The GBM TME impairs γδ T cell function and may facilitate the transition of γδ T cells into a pro-tumoral signature (**Figure 3C**) (58, 135). Though Vγ9Vδ2^+^ γδ T cells are known for their cytotoxic effector function and secretion of tumor-suppressive IFN-γ, they show functional plasticity in the presence of different cytokines. IL-12, IL-18, and type-I IFN induce Th1-like functionality (57, 136), whereas the addition of IL-15 with TGF-β induces Treg-like functionality (58). Furthermore, the combination of IL-6, IL-23, IL-1β, and TGF-β skews Vγ9Vδ2^+^ T cells to Th17-like cells (135). Due to this plasticity, GBM-infiltrated Vγ9Vδ2^+^ T cells may promote rather than suppress tumor growth (**Figure 3C**). TGF-β not only skews Vγ9Vδ2^+^ T cells toward pro-tumoral subtype, but they also dampen the effector function of anti-tumoral functionality of γδ T cells. Rafia et al. showed that after TGF-β treatment, the target-lysing ability of γδ T cells was diminished due to the downregulation of NKG2D and granzyme/perforin expression on γδ T cells (137). In addition, a lymphocyte-depleted TME dampens the antigen-presenting effectiveness of γδ T cells (**Figure 3D**). Although γδ T cells phagocytose and present tumor antigens, there may not be enough CD4 or CD8 T cells in the TME that are primed and activated by this antigen presentation. In addition, TCR stimulation upregulates CXCR6 while downregulating CXCR4, which is required for T cell egress and subsequent localization in the lymphatic organs (138) (**Figure 3E**). γδ T cells in the TME not only phagocytose tumor antigens but are also activated by TCR stimulation, leading to their retention in the tumor. Consequently, γδ T cells cannot spread tumor antigens by egressing out from the tumor and localizing in the lymphatic organs (138).

## 6 Future directions to overcome the limitation of γδ T cells

**Figure 4:**
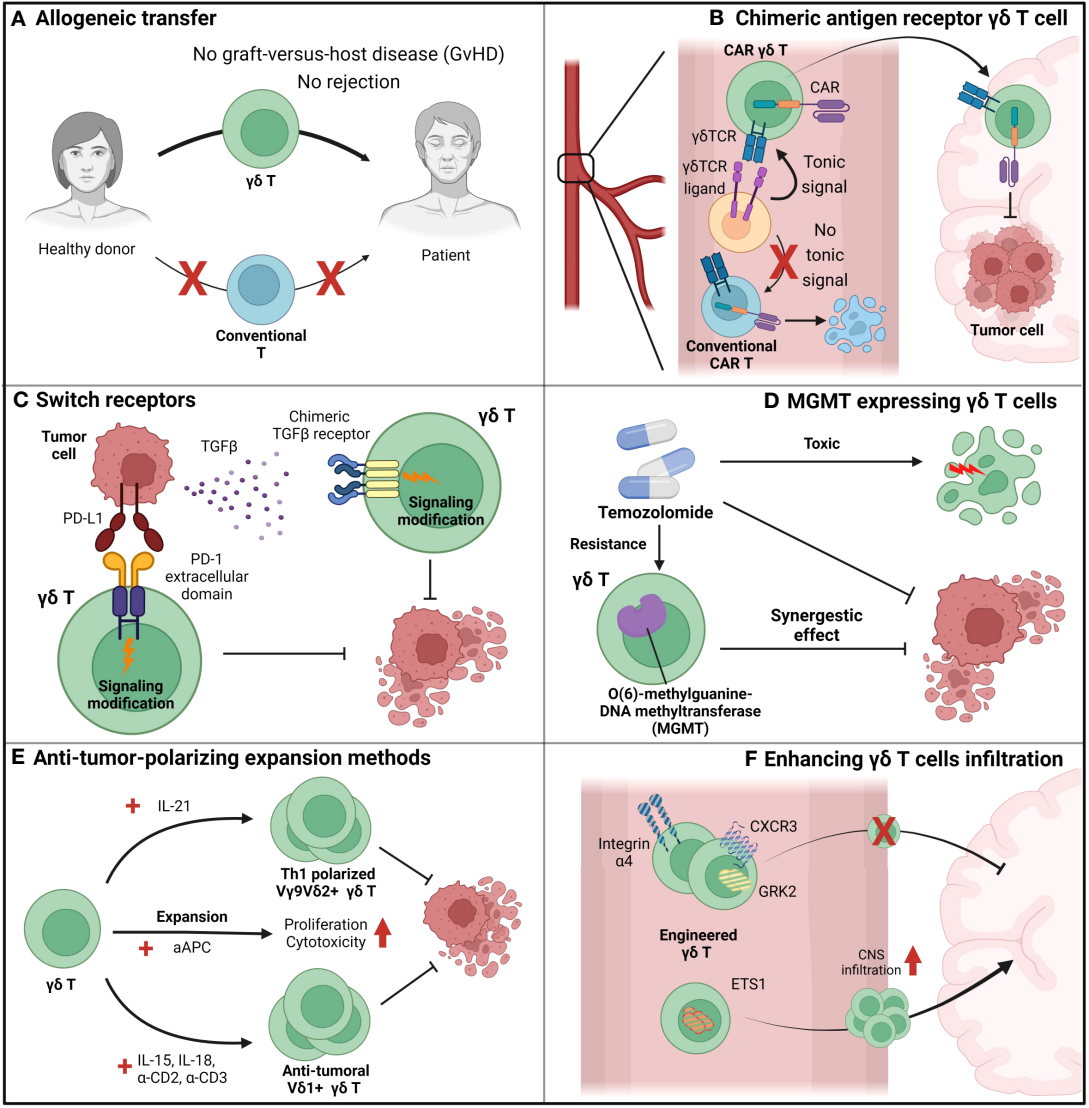
Suggestions to overcome the limiting factors of γδ T cells. **(A)** Allogeneic adoptive cell transfer (ACT) can be beneficial for γδ T cell-based immunotherapy since γδ T cells do not show graft-versus-host diseases (GvHD), in contrast to conventional T cells. By allogeneic ACT of γδ T cells, problems induced by the scarcity of γδ T cells in peripheral blood can be overcome. **(B)** Chimeric-antigen-receptors (CAR)-augmented γδ T cells can be an effective therapeutic option for GBM since they can overcome various issues that arose in conventional T cell-based CAR-T therapy. First, in contrast to conventional T cell-based CAR-T cells, γδ T cell-based CAR-T cells can receive tonic signaling in the peripheral blood, which is critical for CAR-T cell persistence. Next, γδ T cells can lyse GBM tumor cells by its intrinsic γδ TCR, while conventional T cells cannot. Therefore, in contrast to conventional T cell-based CAR-T cells which only target tumor cells by their CARs, γδ T cell-based CAR-T cells can target tumor cells by multiple receptors and block the chance of tumor cells’ evasion of immunosurveillance. **(C)** γδ T cells augmented to express switch-receptors that can exchange the immunosuppressive signaling cues into immune-progressive signaling can overcome the immunosuppressive TME. For example, PD-L1 and TGF- β, well-known anti-inflammatory environmental cues, can be utilized as targets for switch receptors, and γδ T cells expressing switch receptors targeting those factors can sustain their functionality. **(D)** γδ T cells engineered to synergize with other therapy can even increase the therapeutic potential than just a mere combination of two distinct therapy. For example, temozolomide, which suppresses tumor growth but also exerts toxic side-effect on normal immune function, is normally considered a detrimental factor for immunotherapy. However, MGMT-overexpressed γδ T cells, which can overcome the temozolomide-mediated suppression, can synergize with temozolomide, and it is expected that the combination of temozolomide and temozolomide-overcoming γδ T cells can be more effective than just a sum of two single treatment. **(E)** Blocking the plasticity of γδ T cells and polarizing them toward an anti-tumoral population can prevent the skewing of γδ T cells into a pro-tumoral population in the TME. Addition of IL-21 polarizes human Vγ9Vδ2^+^ T cells toward a Th1-like population, and Th1-skewed Vγ9Vδ2^+^ T cells produce pro-inflammatory cytokines and exhibit enhanced cytotoxic roles. If IL-21 mediated polarization could be combined with another expansion protocol with greater expansion efficiency, such as artificial antigen-presenting cell (aAPC)-based methods, the synergistic effect would be dramatic. In addition to Vγ9Vδ2^+^ T cell expansion, another procedure to expand human Vδ1^+^ T cells using IL-15, IL-18, anti-CD2 antibody, and anti-CD3 antibody can efficiently expand and polarize these cells toward an anti-tumoral population. Therefore, with these various procedures to expand and polarize γδ T cells into tumoricidal effectors, γδ T cells could overcome the TME and retain anti-tumoral functionality. **(F)** Augmentation of γδ T cells so that they can cross the BBB can be an effective strategy to transport γδ T cells to the tumor site and increase the number of γδ T cells in the TME. By engineering integrins (e.g., integrin α4), chemokine receptors (e.g., CXCR3 and GRK2), and transcription factors (e.g., ETS1), trafficking of γδ T cells to the central nervous system (CNS) can be modulated.

For successful GBM therapy using γδ T cells, the current limitations of γδ T cells must be addressed and novel therapeutic procedures that fully utilize the benefits of γδ T cells must be devised (**Figure 4**). Rather than expanding patient γδ T cells by zoledronic acid, allogeneic γδ T cell transfer from a healthy donor to the patient is gaining interest (139) (**Figure 4A**). γδ T cells have already proven their safety in allograft transfers, with low risk of graft-versus-host diseases and rejection (131) (**Figure 4A**). With an allograft transfer, global suppression of γδ T cells induced by GBM and chemotherapy will be reduced. In addition to allograft transfers, further engineering of allogeneic γδ T cells can lead to synergistic effects (**Figures 4B-D**). CAR-T cell-based GBM treatment currently shows a modest effect (140), possibly due to the low persistence of CAR-T cells in peripheral blood. It is known that a weak-not high-level of tonic signaling is required for better *in-vivo* persistence and superior antitumor function (141). Anti-EGFRviii CAR-T cells were used for GBM treatment, although this target is not expressed in peripheral blood and cannot provide tonic signaling to T cells (**Figure 4B**). However, the issues caused by the lack of tonic signaling can be resolved by expressing the CAR in human Vγ9Vδ2^+^ γδ T cells, which can receive tonic signaling by γδTCR and have endogenous butyrophilin expression (**Figure 4B**). Introduction of the CAR to γδ T cells provides an additional route by which γδ T cells can target tumor cells, which prevents tumor cells from escaping immune surveillance by antigen loss. In conventional CAR-T cells, which introduce CAR molecules to conventional T cells, tumor cells may escape CAR-T cell surveillance by downregulating the target of the CAR. However, if the CAR is introduced to human Vγ9Vδ2^+^ γδ T cells, tumor cells cannot evade surveillance even after antigen downregulation because γδ T cells can target tumor cells via TCR and other NK receptors. By reducing the chance of tumor cell immune escape, CAR–γδ T cells may represent an improvement over conventional CAR-T cell (**Figure 4B**).

The introduction of engineering expands the opportunities of γδ T cell-based therapy beyond the CAR (**Figures 4C, D**). For example, γδ T cells can be engineered to overcome the immune-suppressive GBM environment. Liu et al. suggested engineering a novel switch receptor that switches the immune-suppressive PD-1 signaling into immune-activating CD28 signaling (142) (**Figure 4C**). Introducing the receptor augmented the anti-tumor immune response of CAR-T cells. In GBM that express PD-L1 (72), engineering γδ T cells by introducing the switch receptor can overcome immunosuppression and may even exploit the suppressive microenvironment. A similar approach to the switch receptor mediation can also be applied to TGF-β to overcome immunosuppression (**Figure 4C**). It is well known that TGF-β is highly expressed in GBM (143), and TGF-β signaling reduces the γδ T cell anti-tumoral immune response by making these cells anti-inflammatory (58). The introduction of a switch receptor that changes the TGF-β signal into other pro-inflammatory signals may help γδ T cells overcome TGF-β-induced immunosuppression. Noh et al. recently introduced a TGF-β-targeting switch receptor that can change TGF-β signaling into IL-7 signaling, and expression of the receptor improved tumor control in the CAR-T-based B-cell lymphoma suppression model (144). Therefore, similar concepts can be applied when designing γδ T cell-based GBM treatment.

Engineered γδ T cells can have synergistic effects when combined with other treatments. Recently, novel genetically engineered human Vγ9Vδ2^+^ γδ T cells were used in a GBM clinical trial (**Figure 4D**). The current standard care therapy for GBM includes temozolomide; however, this treatment affects immune cells, which may lose functionality, because temozolomide does not specifically target tumor cells. In this situation, γδ T cells engineered to express MGMT retain their functionality under temozolomide treatment (145). A clinical trial for GBM treatment using adoptive transfer of human γδ T cells expressing MGMT (ClinicalTrials.gov Identifier: NCT04165941) in combination with temozolomide is currently in progress. In summary, although γδ T cell therapy alone cannot control GBM, it still has therapeutic potential. γδ T cells can overcome current limitations with engineering and combination therapy and may become an effective therapeutic agent for GBM treatment.

Developing novel expansion methods to block the skewing of γδ T cells toward the pro-tumoral population can be an effective and plausible solution for γδ T cell adoptive transfer (**Figure 4E**). Several studies have previously demonstrated procedures to skew γδ T cells toward anti-tumoral populations. For example, the addition of IL-21 helps human Vγ9Vδ2^+^ γδ T cells to produce pro-inflammatory cytokines and exert increased cytotoxicity by irreversibly polarizing Vγ9Vδ2^+^ γδ T cells to express Th1-like signatures (146). This Th1-polarizing condition may show strong synergy with another expansion protocol devised by Choi et al., which uses artificial antigen-presenting cells to expand human Vγ9Vδ2^+^ T cells (147). The expansion strategy proposed by Harmon et al. also showed that addition of IL-15, IL-18, anti-CD2 antibody, and anti-CD3 antibody effectively expanded human Vδ1^+^ T cells and polarized them toward an anti-tumoral population (148). Because the plasticity of γδ T cells in the TME is a major issue that hinders γδ T cell therapy, development of an improved expansion protocol to block this plasticity is crucial for effective γδ T cell therapy.

Engineering γδ T cells to cross the BBB is another effective strategy to increase the infiltration of γδ T cells into GBM (**Figure 4F**). Recent findings from Kendirli et al. show that various factors, ranging from transcription factors to chemokine receptors, regulate T cell migration to the CNS (104). Using genome-wide CRISPR screening, the authors found that knockout of integrin α4, CXCR3, and GRK2 significantly reduced T cell trafficking to the CNS, while ETS1 knockout significantly upregulated T cell trafficking to the CNS. Therefore, modulation of molecules related to T cell trafficking to the CNS in γδ T cells can facilitate infiltration of these cells into GBM.

## 7 Closing remarks

γδ T cells, with their versatile effector functions, have the potential to be a promising therapeutic agent to target tumors. Their ability to target tumor cells via various mechanisms, including γδTCRs, NK receptors, Fc receptors, and death receptors, decreases the possibility of tumor cells evading surveillance. Their ability to produce pro-inflammatory cytokines and spread antigens via direct antigen presentation to the adaptive immune system helps γδ T cells overcome the immunosuppression of the TME and induce optimal anti-tumoral immune responses. Additionally, because they do not recognize MHC molecules and do not risk inducing graft-versus-host disease when transferred from donors to MHC-mismatched patients, γδ T cells can possibly be used in allogeneic adoptive transfer therapy. Therefore, γδ T cells have the potential to be a novel therapeutic agent for GBM, a malignant brain tumor with the highest WHO grade and therefore the worst prognosis. Understanding the immunological signatures of the GBM TME is critical for optimal function of γδ T cells in the GBM TME and subsequent tumor suppression. The immunosuppressive microenvironment, BBB, and MHC-deficient GSCs are the major factors that suppress effective immunotherapy. Although γδ T cells have the potential to overcome some of these limitations, several obstacles still exist, hindering effective therapy and the achievement of successful treatment for GBM. Therefore, for successful γδ T cell–based immunotherapy, it is critical to devise strategies to overcome those limitations. With further studies to determine the signatures of the GBM TME and γδ T cells themselves, in combination with the augmentation of their abilities and improvement of current limitations, γδ T cells can become an innovative therapeutic agent for GBM.

## Author contributions

IK: Conceptualization, Writing – original draft, Writing – review & editing. YK: Conceptualization, Writing – original draft. HL: Conceptualization, Funding acquisition, Supervision, Writing – original draft, Writing – review & editing.
